# The Relevance of the Renin-Angiotensin System in the Development of Drugs to Combat Preeclampsia

**DOI:** 10.1155/2015/572713

**Published:** 2015-04-27

**Authors:** Norikazu Ueki, Satoru Takeda, Daisuke Koya, Keizo Kanasaki

**Affiliations:** ^1^Department of Diabetology and Endocrinology, Kanazawa Medical University, Ishikawa 920-0293, Japan; ^2^Department of Obstetrics and Gynecology, Juntendo University, Tokyo 113-8431, Japan; ^3^Division of Anticipatory Molecular Food Science and Technology, Medical Research Institute, Kanazawa Medical University, Ishikawa 920-0293, Japan

## Abstract

Preeclampsia is a hypertensive disorder that occurs during pregnancy. It has an unknown etiology and affects approximately 5–8% of pregnancies worldwide. The pathophysiology of preeclampsia is not yet known, and preeclampsia has been called “a disease of theories.” The central symptom of preeclampsia is hypertension. However, the etiology of the hypertension is unknown. In this review, we analyze the molecular mechanisms of preeclampsia with a particular focus on the pathogenesis of the hypertension in preeclampsia and its association with the renin-angiotensin system. In addition, we propose potential alternative strategies to target the renin-angiotensin system, which is enhanced during pregnancy.

## 1. Introduction

Preeclampsia refers to a new onset of hypertension (systolic pressure ≥140 mmHg and/or diastolic pressure ≥90 mmHg on two occasions at least 4 hours apart) and either proteinuria (≥300 mg in 24 hours) or end-organ dysfunction (including thrombocytopenia, renal insufficiency, impaired liver function, pulmonary edema, and cerebral symptoms) after 20 weeks of gestation in a previously normotensive woman. Severe hypertension (systolic pressure ≥160 mmHg or diastolic pressure ≥110 mmHg on two occasions at least 4 hours apart while the patient is on bed rest) and symptoms of end-organ injury comprise the severe end of the spectrum of the disease [1]. Worldwide, 10–15% of direct maternal deaths are associated with preeclampsia/eclampsia [2].

Preeclampsia not only is a hypertensive disorder, but also is associated with metabolic defects such as glucose intolerance and dyslipidemia. Preeclamptic women exhibit increased risks of cardiovascular disease (CVD) and type 2 diabetes later in life [3]. During oral glucose tolerance tests, preeclamptic women display higher insulin levels compared with those of normal pregnant women. Preeclampsia is also associated with insulin resistance, and serum free fatty acid levels in preeclamptic women appear to be higher [3, 4]. Insulin resistance, even despite normal glucose levels in early pregnancy, is also associated with preeclampsia onset in later gestational periods [4]. The mechanism explaining such metabolic defects in preeclampsia remains unclear. Therefore, understanding the biology of preeclampsia is important to uncover metabolic defects in pregnancy, a condition associated with potential harmful effects for both the baby and the mother.

Despite such clinical significance, the pathophysiology that leads to preeclampsia remains unknown. Therefore, pathophysiology-based therapies have not yet been established. Hypertension is the major symptom of preeclampsia. However, the molecular mechanisms underlying the onset of hypertension in preeclampsia have not been clearly established. In this review, we analyzed the molecular mechanisms of preeclampsia, with a particular focus on the molecular mechanisms of hypertension in preeclampsia.

## 2. Hypertension in Preeclampsia

Using its worldwide definition, hypertension is the condition of a systolic blood pressure ≥140 mmHg or/and diastolic blood pressure ≥90 mmHg. The pathogenesis of hypertension during pregnancy is not completely understood, and it likely depends on the complex interplay between increased angiotensin II (ATII) activity and mineralocorticoid excess [5], genetic factors [6], endothelial dysfunction [7], neurovascular anomalies [8], and increased sympathetic nervous activity [9].

Clinically, hypertension is the most serious symptom affecting maternal and neonatal health in preeclampsia. In normal human pregnancy, the systolic and diastolic arterial blood pressures decrease slightly due to the reduced total peripheral vascular resistance throughout the course of pregnancy. Normal pregnant women are also known to increase their secretion of aldosterone and to be quite resistant to the pressor effects of ATII [10]. The lowest pressures occur at approximately 28 weeks of gestation; thereafter, the blood pressure fluctuates and tends to return to levels similar to those observed in nonpregnant women [10–14]. Indeed, during pregnancy, left ventricular function is increased as a result of a combination of increased preload, decreased afterload and an increase in intrinsic myocardial contractility that is independent of the loading condition [15]. Plasma volume expansion in pregnant women contributes to protect against placental hypoperfusion [16].

The renin-angiotensin system (RAS) plays an important role in maintaining normal blood pressure, and renin has been recognized as a volume sensor. Low plasma renin activity (PRA) is associated with plasma volume expansion in nonpregnant individuals. The PRA in preeclamptic women is lower compared with that of normal pregnant women [17, 18]. However, such PRA suppression in preeclampsia is unlikely given that the hypertension in preeclampsia is associated with volume-dependent hypertension.

Gant et al. published seminal reports regarding the role of the RAS in the pathogenesis of pregnancy-induced hypertension [19]. In their report, they analyzed the pressor responses to infused ATII that were required to achieve a 20 mmHg rise in diastolic blood pressure in nonpregnant women and 192 pregnant women. Of the latter, 120 women had normal blood pressure throughout the pregnancy, and 72 women subsequently developed pregnancy-induced hypertension. The authors first found that the doses required for a pressor response in normal pregnant women were generally higher than those in nonpregnant women (7.35 ± 0.67 ng/kg/min). The dose required in normal pregnancy increased toward 28 weeks of gestation, and after 30 weeks of gestation, the pressor response dose decreased (8.6 ± 0.76 ng/kg/min at 7–10 weeks of gestation, 14.9 ± 1.1 ng/kg/min at 28 weeks of gestation, and 10.7 ± 0.71 ng/kg/min at 38 weeks of gestation). Compared with nonpregnant women, the amount of ATII required to raise the blood pressure in normal pregnant women was significantly higher. However, the women who later developed pregnancy-induced hypertension were sensitive to the pressor effects of ATII compared with normotensive pregnant women. Women who developed pregnancy-induced hypertension also exhibited resistance to the pressor response early in their pregnancy (12.9 ± 1.1 ng/kg/min at 15–18 weeks of gestation). However, this resistance progressively declined as the pregnancy progressed.

By 23–26 weeks of gestation, there was a clear difference in the pressor response between women with normal pregnancy and women with pregnancy-induced hypertension. The mean required dose of ATII for a pressor response in women who developed pregnancy-induced hypertension progressively declined compared with normotensive pregnant women, and the difference was even greater compared with nonpregnant women. It is important that the sensitivity to ATII between 23 and 32 weeks of gestation was increased in women who developed pregnancy-induced hypertension [19]. Hanssens et al. showed that the lowest ATII levels were found in women with severe pregnancy-induced hypertension [20].

Angiotensin-(1-7) is a bioactive component of the RAS and that displays antagonistic actions of ATII by acting as a modulator of vascular tone and by releasing nitric oxide (NO) and prostaglandins. Angiotensin-(1-7) is generated from ATII by angiotensin-converting enzyme 2 (ACE2) [21–23]. In human studies, plasma angiotensin-(1-7) increases in normal pregnancy and decreases in preeclampsia, and such alternative profiles of ATII products may be relevant to the hypertension and metabolic defects in preeclampsia [21].

In contrast, several groups have suggested that hypertensive disorders result from the presence of agonistic autoantibodies (AAs) that bind to and activate the angiotensin II type 1 (AT1) receptor [24]. Wallukat et al. initially described the presence of AT1-AAs in preeclampsia. Immunoglobulins from the serum of women with preeclampsia stimulated the AT1 receptor and had agonistic activity, but immunoglobulins from normotensive pregnant women had no effect [25]. Immunoglobulin G (IgG) from preeclamptic women contributed to the production of reactive oxygen species by stimulating nicotinamide-adenine dinucleotide phosphate (NADPH) oxidase activity in vascular smooth muscle cells and human trophoblasts [24]. AT1-AAs resulted in increased soluble fms-like tyrosine kinase-1 (sFlt-1) and soluble endoglin (sEng) production from human trophoblasts and placental explants [26, 27]. Irani et al. showed that AT1-AAs induced apoptosis in the placentas of pregnant mice, human villous explants, and human trophoblast cells in culture. The AT1-AAs crossed the placenta and entered the fetal circulation. Finally, the authors showed that the AT1 receptor antagonist losartan diminished intrauterine growth restriction (IUGR) and placental apoptosis, which might be associated with AT1-AAs. The administration of agonistic AT1-AAs obtained from preeclamptic women induced key features of preeclampsia such as hypertension, proteinuria, glomerular endotheliosis, placental abnormalities, and embryonic defects in pregnant mice. These AT1-AA-induced preeclamptic-like symptoms in pregnant mice were attenuated by the AT1 receptor antagonist losartan or neutralizing peptide against AT1-AAs [28]. Wenzel et al. showed that AT1-AAs induced ATII sensitivity in pregnant rats [29].

The evidence from the past studies clearly demonstrates that vasoconstrictive angiotensin receptor signaling activation might be the key to understanding the pathophysiology of hypertension in preeclampsia, despite the suppression of PRA. These findings also indicate that preeclampsia is a hypertensive disorder connected to vasoconstriction induced by vasoactive substances.

## 3. Molecular Regulation of the RAS

**Figure 1:**
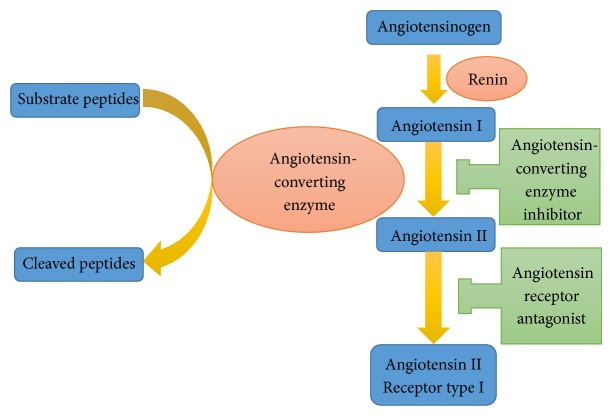
The renin-angiotensin system. Circulating angiotensinogen, derived from the liver, is cleaved by renin to produce angiotensin I. Renin is released into the circulation via the juxtaglomerular cells in the kidney in response to extracellular volume depletion. Angiotensin I is cleaved by angiotensin-converting enzyme into the highly biologically active peptide hormone angiotensin II. Angiotensin II interacts with two major subtypes of cell surface receptor: type I and type II. The actions of angiotensin II are mainly mediated via the angiotensin type I receptor.

Angiotensin-converting enzyme inhibitors (ACEIs) and angiotensin II receptor blockers (ARBs) are commonly used to treat hypertension (Figure 1). The first oral ACEI, captopril, was synthesized in 1975 and approved for clinical use in 1981 by the Food and Drug Administration [30]. Today, RAS inhibitors are considered a major drug classification in the treatment of chronic hypertension. Captopril can be used safely and effectively in managing postpartum hypertension in women with severe preeclampsia [31]. However, the toxicity of these drugs during pregnancy is well documented, and thus, they are contraindicated.

### 3.1. Mechanism

The RAS regulates a variety of physiological/pathological functions, including blood pressure and extracellular fluid volumes [32]. ATII acts directly on vascular smooth muscle cells to produce vasoconstriction, stimulates aldosterone release from the adrenal cortex, and directly suppresses renin release [33]. ATII receptors exist as two subtypes, type 1 (AT1) and type 2 (AT2). The main vasopressor effects are mediated through AT1 receptor binding [34, 35]. AT1 receptors are expressed in multiple organs, including the heart, kidneys, blood vessels, lungs, brain, liver, and adrenals [36].

ACEIs exert their antihypertensive effects by both inhibiting the conversion of angiotensin I (ATI) to ATII and degrading bradykinin, a potent vasodilator [37]. Consequently, ACEIs also decrease the ATII-induced production of aldosterone. ACEIs enhance the distribution of blood flow to the kidneys, heart, and brain without altering cardiac output [38]. Despite the reduction in blood pressure, ACEIs have little effect on heart rate [33, 37]. ACEI treatment results in a reduced total peripheral resistance, and there is little change in the pulmonary capillary wedge pressure [33].

### 3.2. The Mechanisms of Placentation by Oxygen Levels

The placenta is an essential organ that provides oxygen, water, carbohydrates, amino acids, lipids, vitamins, minerals, and other nutrients to the fetus. At the same time, the placenta is responsible for removing carbon dioxide and other waste products from the fetus [39]. Fetoplacental vasculogenesis begins on day 21 after conception with the differentiation of angioblasts, and angiogenesis begins on day 32 after conception and continues until 25 weeks of gestation [40, 41]. Trophoblast cells undergo proliferation and differentiation into cytotrophoblasts (CTBs) and extravillous trophoblasts (EVTs). CTBs penetrate the layer of syncytiotrophoblasts (SCTs) to form columns of EVTs. EVTs invade the decidua and remodel maternal blood vessels. This process produces dilated uterine arterioles that are unresponsive to maternal vasoconstrictors. Consequently, the placental circulation is independent of blood pressure regulation mediated by maternal factors [39, 42].

The embryo and human placenta develop in a low-oxygen environment during the first trimester in the absence of the maternal circulation, resulting in a reduced risk of free radicals for the early conceptus. The hypoxic environment during the first trimester is believed to facilitate trophoblast invasion, although some authors have suggested that the hypoxic conditions inhibit trophoblast invasion. The low-oxygen condition induces hypoxia-inducible factor (HIF)-1*α* and transforming growth factor- (TGF-) *β*3 [43]. TGF-*β*3 inhibits trophoblast differentiation toward an invasive phenotype in first-trimester human placental explants. HIF-1*α* mRNA levels are high in placental trophoblasts between 5 and 8 weeks of gestation, and then they fall steeply at approximately 10 to 12 weeks of gestation, when the intraplacental oxygen concentration rises three-fold compared with the early first trimester because the maternal intervillous circulation becomes established [44]. The increasing placental pO_2_ reduces HIF-1*α* expression and consequently downregulates trophoblast TGF-*β*3 expression. As a result, the downregulation of TGF-*β*3 triggers trophoblastic differentiation into invasive EVTs, which invade deep into the maternal uterus to remodel maternal spiral arteries. Abnormalities in TGF-*β*3 expression are associated with preeclampsia [43–47].

**Figure 2:**
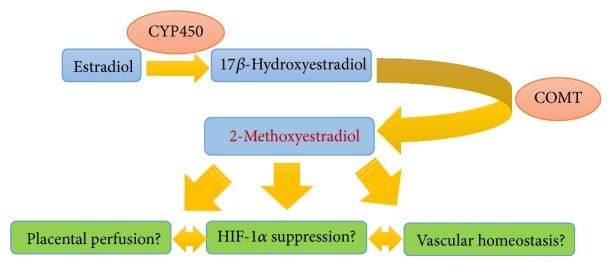
The putative role of the catechol-O-methyltransferase (COMT)/2-methoxyestradiol (2ME) system in pregnancy. In normal pregnancy, 2ME may have a role in regulating hypoxia-inducible factor (HIF)-1*α* in diverse ways. In preeclampsia, low COMT/2ME levels may induce the accumulation of HIF-1*α*, leading to vascular defects, placental hypoxia, and inflammatory responses in the placenta. Such a response may induce placental defects. CYP450: cytochrome P450.

The antitrophoblast invasion roles of HIF-1*α* overexpression and TGF-*β*3 expression have largely been corroborated by experiments using the endogenous HIF-1*α* inhibitory molecule 2-methoxyestradiol (2ME). 2ME is a metabolite of estradiol and is generated by catechol-O-methyltransferase (COMT) through the intermediate metabolite 2-hydroxyestradiol (Figure 2). 2ME induces the invasion of cytotrophoblasts specifically under low-oxygen conditions (2.5% O_2_), and the invasive effects of 2ME under low-oxygen conditions are associated with a decrease in the expression of HIF-1*α* and TGF-*β*3. Under low-oxygen exposure conditions with 2ME, trophoblasts differentiate with an invasive phenotype and migrate through Matrigel. Neither low-oxygen tension nor 2ME alone stimulates invasion by trophoblasts. The invasion of trophoblasts into the myometrium is regulated by the oxygen concentration gradient between the placenta, decidua, and uterine wall. It is hypothesized that trophoblasts lose their invasive phenotype as they invade deeper into the uterine wall and encounter higher oxygen levels [48]. 2ME is elevated during the third trimester of normal human pregnancy. However, the level of 2ME is significantly lower in women with severe preeclampsia [49–51].

### 3.3. The Relevance of the RAS in Placentation

Hypoxia (3% oxygen) enhances vascular endothelial growth factor- (VEGF-) stimulated human placental artery endothelial cells. VEGF is the principal mediator of angiogenesis, and the major transcriptional activator of the VEGF gene is HIF-1*α* [44, 46, 52, 53]. The activation of AT1 receptors increases VEGF in human umbilical vein endothelial cells [54]. VEGF induces endothelial proliferation, acting through two tyrosine kinase receptors, VEGFR1 (FLT1) and VEGFR2 (KDR). Moreover, the increased endothelial proliferation is suppressed by the administration of an AT1 receptor blocker or VEGF antagonist [54–57].

In contrast, AT2 receptor expression is downregulated in maternal tissues in pregnancy because activation of the AT2 receptor inhibits VEGF [58, 59]. AT1-AAs have a direct effect on cell migration and angiogenesis through activating the AT1 receptor and are positively correlated with VEGF levels in endothelial ovarian cancer patients. ATII-induced tumor cell invasion, angiogenesis, and peritoneal dissemination are blocked by AT1 receptor antagonists [60, 61]. AT1 receptor-regulated cell migration is also likely to be crucial for placentation, because AT1 receptor-deficient female mice exhibited placental defects that included a thickened decidual layer and abolished giant cell and labyrinth [62].

Trophoblast-secreted plasminogen activator (PA) activates plasminogen. PA also remains in an active form bound to PA receptors on the cell membrane [63]. Plasminogen is converted into active plasmin through the action of urokinase-type PA (uPA). uPA is implicated in the control of implantation due to its role in extracellular matrix degradation. Plasmin, a serine protease, results in the activation of metalloproteinases. Metalloproteinases and plasmin appear to be the key enzymes in trophoblast invasion [63, 64]. The activity of uPA is regulated by plasminogen activator inhibitor-1 (PAI-1) [65]. The activation of AT1 receptor by ATII stimulates PAI-1 production by human trophoblasts in a time- and dose-dependent manner. Similarly, hypoxia increases the expression of PAI-1 mRNA and protein [66]. The activation of AT1 receptors increases cell proliferation and decreases trophoblast invasion by increasing the TGF-*β*1 and PAI-1 levels [65]. TGF-*β*1 increases the synthesis of PAI-1 [67, 68]. PAI-1 levels are significantly increased in the plasma and placenta of preeclamptic women compared with normal pregnant women. Increased PAI-1 is associated with shallow trophoblast invasion and hypercoagulation [65, 69]. AT1-AAs activate AT1 receptors on human trophoblasts resulting in increased expression of the PAI-1 protein. Then, AT1-AA-induced PAI-1 overproduction is blocked by the AT1 receptor antagonist losartan [65, 69–71].

### 3.4. The RAS and Fetal Development

The RAS is active during fetal development and has an important role in controlling the umbilical-placental circulation, fetal blood pressure, and cardiovascular function through ATII [72, 73]. Animal studies also demonstrated that ATII induces cell growth through AT1 receptor, which is essential for the development of the fetal kidney [74].

In humans, all of the components of the RAS are expressed as early as 5 weeks of gestation in human embryos [75–77]. One study found the expression of only AT2 receptors in the kidneys of 17–26 weeks of gestation in human fetuses [78]. In humans, nephrogenesis begins in 5 weeks of gestation and ceases by approximately 36 weeks of gestation, by which time approximately 1 million nephrons per kidney are present. The fetus starts to produce urine by 9–12 weeks of gestation [79, 80]. The GFR increases progressively up to 34 to 36 weeks of gestation and matures rapidly in the early postnatal period [81, 82]. In humans, mutations in genes that encode renin, angiotensinogen, ACE, and AT1 were found to be associated with the autosomal recessive disease renal tubular dysgenesis. Renal tubular dysgenesis causes oligohydramnios, which leads to the Potter sequence and skull ossification defects [83].

In AT1 receptor-deficient pregnant mice, the number of live newborns is significantly reduced by the placental malformation in 30% of all uteroplacental units. Within the malformed uteroplacental units, normal embryonic structure is not observed [62]. The human-angiotensinogen transgenic female mice (hAG^+/+^) mated with the human-renin transgenic male mice (hRN^+/+^) display hypertension in late pregnancy due to secretion of human renin from the fetal side into the maternal circulation and exhibit placental abnormalities, maternal cardiac hypertrophy, proteinuria, and IUGR [84, 85]. However, hAG^+/+^ females lacking ATII receptor type 1a (mAT1a) mated with hRN^+/+^ male mice (pregnant hAG^+/+^/mAT1a^−/−^) have a normotensive phenotype, and the placental abnormalities, maternal cardiac hypertrophy, and IUGR are ameliorated [85]. Similarly, pregnant hAG^+/+^ mice administered AT1 antagonist (5 mg/kg/day) on gestation days 18 and 19 exhibit improved hypertension and IUGR [85]. In brief, the lack of maternal AT1 might not affect fetal development while having a positive effect on maternal hypertension. ARB might therefore be an effective medication for pregnancy-induced hypertension.

### 3.5. The Use of ACEIs or ARBs in the First Trimester

Cooper et al. studied a cohort of 29,507 infants enrolled in Tennessee Medicaid whose mothers had no evidence of maternal diabetes. The risk of major congenital anomalies among 209 infants with exposure to ACEIs in the first trimester alone was compared with the risk in 202 infants with exposure to other antihypertensive medications (excluding ARB) in the first trimester alone and 29,096 infants with no exposure to antihypertensive drugs at any time during pregnancy. They found that 7.1% of the infants with exposure to ACEIs in the first trimester alone had major congenital anomalies after adjusting for confounders, which was 2.7-fold higher than infants with no exposure to antihypertensive drugs at any time during pregnancy. And they found a significantly increased risk of cardiovascular (risk ratio, 3.72; 95% confidence interval (CI), 1.89–7.30) and central nervous system (CNS; risk ratio, 4.39; 95% CI, 1.37–14.02) malformations [86]. However, some women with undiagnosed diabetes were not excluded, and also prepregnancy body mass index was not controlled in this study, despite maternal diabetes, overweight, and obesity being associated with congenital malformations [87–89].

The association between maternal use of ACEIs or other antihypertensive medications in the first trimester and congenital anomalies among infants was explored in a record linkage study performed through the Swedish Medical Birth Register [90]. In this study, women treated with antihypertensive medications had an increased risk of preterm delivery, placental abruption, caesarean delivery, and labor induction. Cardiovascular defects occurred with adjusted odds rate of 2.59 (95% CI, 1.92–3.51). However, the results were similar for the use of an ACEI or ARB during the first trimester compared with other antihypertensive medications, particularly beta blockers [90].

Outcomes were studied in 91 pregnant women in which the mother took either an ACEI or ARB during early pregnancy. Of the 71 pregnancies in women taking an ACEI, six babies (10.2%) had developmental anomalies: one small ventricular septal defect (1.4%), one mild sensorineural deafness (1.4%), one mild microcephaly (1.4%), one hypospadias (1.4%), one umbilical hernia (1.4%), and one mild congenital hypotonia (1.4%). In the 20 pregnancies (21 babies) in women who conceived while taking an ARB, two developmental defects were identified: one inguinal hernia (5%) and one craniosynostosis with tower skull (5%). This study did not find any convincing excess of congenital anomalies in women taking an ACEI or ARB in early pregnancy [91].

Porta et al. reported the experience from the DIRECT (Diabetic Retinopathy and Candesartan Trials) study. A group of 615 women with type 1 diabetes and no retinopathy and 813 women with type 1 diabetes and mild to moderately severe nonproliferative retinopathy were randomized to either candesartan (32 mg/day) or placebo. A total of 42 women taking candesartan and 45 taking a placebo became pregnant and discontinued treatment 0 to 8 weeks after their last menstrual period. Pregnancy outcomes were found to be similar for both groups, with no increase in the rate of malformation in neonates. There were two stillbirths and two “sick babies” in the candesartan group; there were one stillbirth, eight “sick babies,” and one ventricular septal defect in the placebo group. This study was a randomized trial, and all of the women had diabetes and were normotensive. Although no woman continued candesartan after eight weeks of gestation, the average length of time the women were exposed to candesartan is not known [92].

Li et al. conducted a population-based retrospective cohort study of 465,754 pregnant women in northern California and their live-born offspring. Women who used an ARB during pregnancy were excluded from the study. The controls were two cohorts composed of women who had diagnoses of hypertension but had not used any antihypertensive drug during pregnancy and women who had neither a diagnosis of hypertension nor a record of any prescription for an antihypertensive drug. After adjustment for maternal age, ethnicity, parity, and obesity, the rate of congenital heart defects among the offspring of women who used an ACEI in the first trimester (15/381 (3.9%)) was similar to the rate among the offspring of women who used other antihypertensive drugs (28/1090 (2.6%)). This study found a similarly elevated risk of congenital heart defects in the offspring of women who had used either ACEIs or other antihypertensive drugs or who had hypertension and did not use antihypertensive drugs during the first trimester of pregnancy (708/29735 (2.4%)). The authors suggested that it is likely that underlying hypertension increased the risk of congenital heart defects in the offspring [93].

### 3.6. The Use of ACEIs or ARBs in the Second or Third Trimester

Using captopril (2.8–3.5 mg/kg) in pregnant sheep during late pregnancy (119–133 days of gestation—term is 147 days) reduced the maternal blood pressure transiently for 2 h. However, the fetal blood pressure remained reduced for up to 2 days [94]. The pressor response was evoked by an intravenous bolus of ATI (167–426 ng/kg). When the blood pressure was again stable, captopril was again given to the ewe. Basal blood pressures had fallen in all ewes and fetuses by 10 min after captopril administration. The risk of stillbirth was significantly elevated, and 7 of the 8 ewes produced stillborn lambs. The administration of captopril (3.3 mg) to late-gestational-age rabbits (24–28 days of gestation—term is 31 days) resulted in a 37% stillbirth rate compared with 6% in saline control rabbits. The inhibition of AT II synthesis reduced uterine blood flow and increased fetal mortality [94]. Harewood et al. investigated fetal deaths were associated with the decrease of maternal blood pressure, and the fetal mortality was likely secondary to the direct effect of enalapril (7.5 mg/day) on the fetal RAS rather than to the effects of placental ischemia. Postmortem examination of the dead fetuses revealed no fetal anomalies [95]. Some animal experiments suggest that the use of ACEIs in the second or third trimester leads to decreased uteroplacental blood flow, low birth weight, fetal hypotension, preterm delivery, and fetal death.

In human studies, the most commonly reported adverse effects of ACEIs or ARBs taken in the second or third trimester include suppressed fetal renal function and reduced urine output, which lead to oligohydramnios with fetal anuria, neonatal hypotension, renal failure, and hypocalvaria [96–99].

According to the above reports, fetal exposure to ACEIs or ARBs in the first trimester only is not likely to be associated with fetal anomalies. However, fetal exposure to ACEIs or ARBs during the second or third trimester is associated with fetal anomalies. ACEIs and ARBs should be avoided in women who are or may become pregnant.

## 4. Perspective: What Alternative Therapies Are Available?

Theoretically, suppression of the RAS would be reasonable in preeclampsia treatment; however, due to the anomalies reported with the use of drugs that suppress the RAS, we cannot recommend this class of drugs for the treatment of preeclamptic women. For alternative strategies to target the RAS, we have provided several ideas to combat preeclampsia that are associated with potential RAS inhibition.

### 4.1. Rest and Physical Activity

Preeclampsia has been believed as the condition associated with depletion of plasma volume [100]. Placental perfusion is likely decreased by exercise and the bed rest in pregnant women displays increased placental perfusion, the hypothetical condition associated with the onset of preeclampsia [101]. Indeed the bed rest has been considered in many pregnancies to prevent or treat a variety of conditions, such as spontaneous abortion, preterm labor, fetal growth retardation, edema, chronic hypertension, and preeclampsia. However in the treatment of preeclampsia, the effects of bed rest are still questionable in its view [102]. Also there might be risks associated with rest, such as deep vein thrombosis and pulmonary embolism [103]. In WHO, advice rest at home and strict bedrest are not recommended for prevention or improving pregnancy outcomes in women with preeclampsia [104].

Renin-angiotensin system is altered by physical exercise. In the patient with heart failure, physical exercise has been shown to reduce sympathoexcitatory process by reducing oxidative stress, increasing nitric oxide (NO), and reducing ATII [105]. Epidemiological data suggested that the physical activity either occupational or leisure-time was associated with the reduction in the incidence of preeclampsia [106]. However, it is not concluded whether interventional physical exercise is of benefit to the disease progression of preeclampsia since very limited information is only available in the literature [106–108].

### 4.2. Antioxidants

Oxidative stress, which diminishes the bioavailability of NO, has been proposed to increase the risk of essential hypertension and preeclampsia [109, 110]. In animal studies, ATII increases vascular superoxide production, which reduces the biologic activity of endothelium-derived NO [111]. In humans, ATII blockade attenuates endothelium-dependent forearm vasodilation, and vitamin C improves the endothelial impairment [112]. Some authors have assessed the effects of antioxidant supplementation with vitamins C and E on the risk of preeclampsia. Pregnant women between 9 and 16 weeks of gestation or between 14 and 22 weeks of gestation were randomly assigned to daily supplementation with 1000 mg of vitamin C and 400 IU of vitamin E or placebo until delivery. Supplementation with vitamins C and E initiated at 9 to 16 weeks of gestation or at 14 and 22 weeks of gestation until delivery did not reduce the rate of preeclampsia [110, 113]. Previous studies have suggested that low maternal serum 25-hydroxy vitamin D (25(OH)D) levels increase the risk of preeclampsia and that vitamin D supplementation lowers this risk [114]. Compared with normotensive individuals with sufficient 25(OH)D levels (≥30.0 ng/mL), individuals with 25(OH)D insufficiency (15.0 to 29.9 ng/mL) and deficiency (<15.0 ng/mL) had higher circulating ATII levels. Moreover, compared with individuals with sufficient vitamin D levels (145 mL/min/1.73 m^2^), those with vitamin D deficiency (115 mL/min/1.73 m^2^) had significantly slowed renal plasma flow responses to infused ATII [115].

### 4.3. Anticoagulation Therapies

Preeclampsia is associated with thrombocytopenia, disseminated intravascular coagulation, and platelet aggregation. In addition, abnormal placental development results in placental ischemia. In vitro, aspirin suppresses ATII-mediated AT1R and VEGF expression in HMVECs [116]. Previous studies suggested that daily administration of antiplatelet agents, particularly low-dose aspirin beginning as early as the second trimester, had benefits for the prevention of preeclampsia. No harmful effects were identified, but the long-term evidence is limited [117, 118]. CLASP, a multicenter trial in which 9364 women were randomly assigned 60 mg aspirin daily or a matching placebo, suggested that low-dose aspirin did not prevent the incidence of proteinuric preeclampsia [119]. Furthermore, Kyle et al. suggested a contrasting result and showed that the administration of low-dose aspirin (60 mg/day) did not prevent preeclampsia when initiated in 28 weeks of gestation in ATII-sensitive women [120]. In 2014, the US Preventive Services Task Force (USPSTF) recommended the use of low-dose aspirin (81 mg/day) as a preventive medication after 12 weeks of gestation in women who are at high risk for preeclampsia [117].

### 4.4. COMT and 2ME

Kanasaki et al. reported that pregnant mice deficient in COMT showed a preeclampsia-like phenotype resulting from the absence of 2ME, a natural metabolite of estradiol that is elevated during the third trimester of normal human pregnancy [49, 51]. COMT is a catabolic enzyme involved in the degradation of a number of bioactive molecules such as catecholamines and catecholestrogens. Estradiol is metabolized by cytochrome p450, and the resultant 17-hydroxyestradiol is a substrate for COMT, which converts 17-hydroxyestradiol into 2ME as a rate-limiting step in estrogen breakdown. The concentration of maternal 2ME in the circulation immediately increases during normal pregnancy; however, the levels of COMT and 2ME are significantly lower in women with severe preeclampsia [49–51]. We know that hydralazine is widely used in the treatment of preeclampsia. However, hydralazine inhibits placental COMT activity [121]; thus, physicians should be careful when using it.

2ME has been shown to ameliorate all preeclampsia-like features without toxicity in* Comt(−/−)* pregnant mice and to suppress placental hypoxia, HIF-1*α* expression, and increased sFLT-1 expression [49]. Furthermore, 2ME may directly function as a vasodilator and inhibit vasospasm in pregnant women [122]. 2ME suppresses the AT1 receptor in vascular smooth muscle cells [123]. Finally, as described above, 2ME ameliorates the restricted trophoblast invasion in preeclampsia via suppression of HIF-1*α*. 2ME (Panzem) is now in development as an anticancer drug that induces HIF-1*α* suppression (phase II clinical trials) [124–126]. Therefore, 2ME is available as a drug that is orally available. If 2ME is safe to use in pregnant women, it could be a therapeutic drug for preeclampsia.

Alternatively, nutritional intervention also can enhance the COMT-2ME system during pregnancy. Elevated homocysteine levels might be associated with an increased risk of preeclampsia, and homocysteine is converted into S-adenosyl homocysteine (SAH), a potent COMT inhibitor. Folic acid acts to remethylate homocysteine, converting homocysteine back into methionine [127–129]. Supplementation with folic acid during the second trimester is associated with a reduced risk of preeclampsia [130]. COMT may be suppressed by various endogenous and exogenous molecules, including polychlorinated biphenols (PCBs), dioxin, mercury, and SAH. Therefore, epidemiologically, avoiding exposure to such environmental COMT suppressors would be relevant to the design of any antipreeclampsia strategy.

## 5. Conclusions

The pathophysiology of preeclampsia is not completely understood. Many hypotheses, theories, and models are associated with preeclampsia, many of which have focused on the abnormalities of angiogenesis. However, few investigators have tested whether the proposed hypertensive mechanisms are relevant to the pathophysiology of hypertension in preeclamptic women. Careful attention should be paid when designing strategies to combat preeclampsia based on the pathogenesis known in both human disease and animal models. Further research would be required to obtain deeper insight into the pathogenesis of preeclampsia by designing experiments to test each hypothesis in relation to human disease.
